# Profiling of Fungal Diversity and Fermentative Yeasts in Traditional Chinese *Xiaoqu*

**DOI:** 10.3389/fmicb.2020.02103

**Published:** 2020-08-31

**Authors:** Chunxiao Wang, Jiadai Tang, Shuyi Qiu

**Affiliations:** ^1^Province Key Laboratory of Fermentation Engineering and Biopharmacy, School of Liquor and Food Engineering, Key Laboratory of Plant Resource Conservation and Germplasm Innovation in Mountainous Region (Ministry of Education), Guizhou University, Guiyang, China; ^2^Department of Liquor Making Engineering, Moutai Institute, Renhuai, China

**Keywords:** rice wine, baijiu, high throughput sequencing, *Saccharomyces cerevisiae*, *Wickerhamomyces anomalus*

## Abstract

To increase the safety and quality of baijiu and rice wine in China, controlling the use of traditional *Xiaoqu* by studying the beneficial yeasts present has recently been considered. The fungal diversity of six Chinese *Xiaoqu* including five traditional and one commercial samples was investigated to screen fermentative yeasts with low yields of higher alcohols. A high throughput sequencing approach detected fifteen fungal species with relative abundance higher than 1%, and displayed dissimilarities of fungal diversity among *Xiaoqu* samples. The 15 fungal species were composed of 11 filamentous fungi with *Rhizopus arrhizus* as the most common specie and four yeast species, containing *Hyphopichia burtonii*, *Saccharomyces cerevisiae*, *Saccharomycopsis fibuligera*, and *Saccharomycopsis malanga*. Classic culture-dependent approaches, including 5.8S-ITS-RFLP analysis and D1/D2 sequencing of the 26S rRNA gene, identified nine yeast species in the five traditional Chinese *Xiaoqu*. In addition to the four yeast species also detected by high throughput sequencing approach, the other five yeast species isolated were *Clavispora lusitaniae*, *Cryptococcus neoformans*, *Komagataella pastoris*, *Trichosporon asahii*, and *Wickerhamomyces anomalus*. Further micro-fermentations of rice wine were performed using 19 single yeast isolates, and after the fermentation of rice wine, higher alcohols and ethanol were analyzed by gas chromatography. Two yeast strains, *Saccharomyces cerevisiae* FBKL2.8022 and *Wickerhamomyces anomalus* FBKL2.8023, were found to have low yields of higher alcohols and could produce 11.70%vol and 7.10%vol ethanol separately. This study for the first time, to the best of our knowledge, explored the fungal resources in traditional *Xiaoqu* from different regions of Guizhou, China. The screened *S. cerevisiae* and *W. anomalus* strains could be used to establish specific starters to promote the standardization of the production of baijiu and rice wine.

## Introduction

*Xiaoqu* is a traditional fermentation starter in China that utilizes rice as a raw material (Gou et al., 2015; Liu and Sun, 2018). *Xiaoqu* is named as such due to its smaller size compared to Daqu, and is similar to Nuruk in Korea and Koji in Japan in terms of their use in producing rice wine (Jin et al., 2017; Cai et al., 2018). *Xiaoqu* is widely used in South China to produce rice, herbal, chi aroma baijiu and rice wine (Liu and Sun, 2018; Chen et al., 2019). Guizhou, a southwestern province, is famous for sauce aroma baijiu as well as traditional rice wine and *Xiaoqu*. *Xiaoqu* as a fermentation starter, impacts the flavor and quality of baijiu and rice wine (Jin et al., 2017; Zhang et al., 2018; Chen et al., 2019). Therefore, investigating the fungal diversity and beneficial yeast in traditional Guizhou *Xiaoqu* is necessary to improve the quality of baijiu and rice wine in China.

**FIGURE 1:**
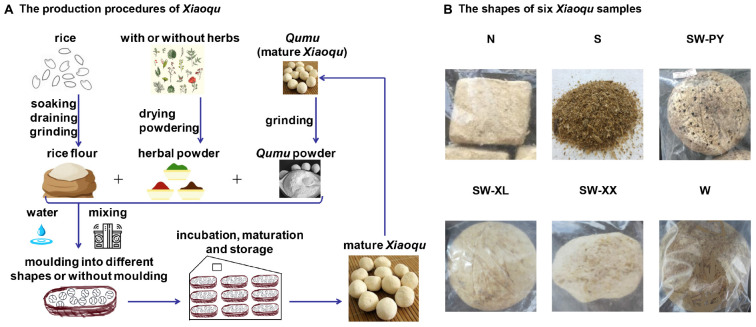
The production processes of *Xiaoqu* and the shapes of six *Xiaoqu* samples. **(A)** The production processes of *Xiaoqu*. The primary raw material is rice, and others also include cereal or Chinese herbs. Rice is soaked in water for 2–3 h then drained and ground into rice flour. The rice flour with or without herbal powder is mixed with *Qumu* powder and water (60%), then manually molded into different shapes (sphere, quadrate, cake-like, etc.). *Sanqu* was produced using an automatic starter-making disk machine without molding step (Wang et al., 2018). *Xiaoqu* was stored in a Qu house, namely a cultivation room, for approximately 4 days at 28–34°C. Mature *Xiaoqu* is obtained after drying, maturation, and storage. **(B)** The shapes of six *Xiaoqu* samples. *Xiaoqu* samples were labeled as N, S, SW-PY, SW-XL, SW-XX, and W, corresponding to their collection from northern, southern, southwestern (Panzhou country), southwestern (Liguan of Xingren country), southwestern (Xiaojiawan of Xingren country), and western region of the Guizhou province.

The production methods of *Xiaoqu* stem from ancient times in China (Jin et al., 2017). Traditionally, *Xiaoqu* is produced from rice flour inoculated with *Qumu*, which is mature *Xiaoqu* and has good amylolytic and fermentation properties (Figure 1). *Qumu* contains different fungi and bacteria, which originate from raw materials, production microhabitats, and the inoculation of molds and yeasts (Gou et al., 2015; Cai et al., 2018). Commercial *Xiaoqu* is generally inoculated with a pure culture of beneficial molds and yeasts, that are screened from traditional *Qumu*. However, some Guizhou distilleries still use homemade *Qumu*, known as traditional *Xiaoqu*, to maintain their distinctive flavors. Traditional *Xiaoqu* contains rich natural microbial resources, especially yeasts and molds that play key roles (Wu et al., 2017). A typical example is *Rhizopus* Q303, a mold screened from traditional *Xiaoqu* by the Guizhou Provincial Light Industry Scientific Research Institute in 1977 that has been widely applied in baijiu and rice wine production due to its strong amylolytic ability (Fu, 1987). Investigating the fungal diversity of traditional *Xiaoqu* is beneficial for the isolation of advantageous molds and yeasts, which traditionally relied on laborious culture-dependent approaches and has only recently been upgraded to high throughput sequencing techniques. The advantageous molds and yeasts isolated from traditional *Xiaoqu* include *Rhizopus oryzae*, *Rhizopus peka*, *Rhizopus chinensis*, *Absidia* sp., *Aspergillus oryzae*, *Mucor rouxianus*, *Saccharomyces cerevisiae*, *Saccharomycopsis fibuligera* and *Wickerhamomyces anomalus* (synonyms *Hansenula anomala*, *Pichia anomala*) (Wu, 2004; Li and Fang, 2007; Jin et al., 2017). Gou et al. (2015) identified two primary fungal species of traditional *Xiaoqu* from Sichuan province, *R. oryzae* and *S’copsis fibuligera*, using 18S-amplicon high throughput sequencing techniques. Four species *Rhizopus stolonifer*, *Cunninghamella bertholletiae*, *S. cerevisiae*, and *Candida fennica* were identified in traditional *Xiaoqu* from Hubei and Sichuan provinces by 18S-amplicon high throughput sequencing techniques (Wu et al., 2017). Cai et al. (2018) detected eight amylolytic species of *Rhizopus*, *Aspergillus*, *Mucor*, *Neurospora*, and *Saccharomycopsis*, and four ethanol-producing species of *Saccharomyces*, *Pichia* and *Candida* in traditional *Xiaoqu* from Hubei, Jiangsu, Zhejiang, Guangxi, Sichuan, and Guizhou provinces using ITS-amplicon high throughput sequencing techniques. However, limited information is currently available regarding fungal diversity and beneficial fungi in traditional Chinese *Xiaoqu*.

Higher alcohols, such as isobutanol and isoamyl alcohol, at moderate levels contribute to the flavor of many alcoholic drinks, whereas excess levels may be a health hazard (Lachenmeier et al., 2008; Styger et al., 2011; Zhu et al., 2016), and thus, the amount present in alcoholic drinks should be controlled (Jin et al., 2017; Li et al., 2017). Higher alcohols are secondary metabolites of yeast during alcoholic fermentation, and are primarily synthesized by the catabolism of amino acids, also known as the Ehrlich pathway (Hazelwood et al., 2008). Studies from China have involved the screening of several yeast strains with low yields of higher alcohols such as *S. cerevisiae* (Strain code JH301, AY15-BAT2, and ARTP5, etc.) obtained by natural separation, haploid preparation, or strain mutagenesis (Wang et al., 2019a). To the best of our knowledge, no yeasts with low yields of higher alcohols have previously been isolated from *Xiaoqu*. Traditional rice wine production methods depend on the individual’s operation experience and lack control and standardization, and thus, commonly cause headaches or hangovers due to excess amounts of higher alcohols and methanol present (Chung et al., 2012; Yan et al., 2012). Sake yeast (*Saccharomyces sake*) strains with favorable traits, such as high fermentation ability (Kyokai Strain No. 6) and non-urea-producing (Kyokai Strain No. 1901), have been used for sake brewing since 1895, demonstrating the role of beneficial yeast on sake fermentation and quality improvement (Ohya and Kashima, 2019). Therefore, using the rich fungal resources in traditional Chinese *Xiaoqu* to screen fermentative yeasts with low yields of higher alcohols is advantageous.

To promote standardization of the production of baijiu and rice wine from *Xiaoqu*, beneficial molds and yeasts with specific industrial use have been a focus for producing starters. The aim of this study was first to collect traditional *Xiaoqu* from different regions in the Guizhou province of China and analyze their fungal resources. Secondly, this study aims to screen yeasts with ethanol-producing abilities and low yields of higher alcohols on the basis of the rich fungal resources in traditional folk *Xiaoqu*. Fungal diversity in *Xiaoqu* samples was investigated via high throughput sequencing techniques. Yeasts were isolated and identified through classic culture-dependent approaches. Micro-fermentations inoculated with a single isolated yeast species were performed to produce rice wine. The ethanol and higher alcohol contents after rice wine fermentation were analyzed, and fermentative yeasts were screened for their fermentation performance and higher alcohol yields.

## Materials and Methods

### *Xiaoqu* Samples

*Xiaoqu* samples were collected from six distilleries in different regions of Guizhou of China in September 2017, with five samples being traditional *Xiaoqu* and one being commercial *Xiaoqu*. The six samples were labeled N, S, SW-PY, SW-XL, SW-XX, and W according to the location of the distilleries (Figure 1 and Supplementary Table S1). *Xiaoqu* samples were maintained on ice during transportation, and were stored at −20°C for high throughput sequencing analysis and 4°C for yeast isolation.

### High Throughput Sequencing Analysis

Six *Xiaoqu* samples (more than three blocks in each sample) were sent to Biomarker Technologies Corporation (Beijing, China) for DNA extraction and further high throughput sequencing. DNA was extracted from 0.25 g *Xiaoqu* samples using a PowerSoil^®^ DNA Isolation Kit (MO BIO Laboratories, Inc., Carlsbad, California). Primers of VnF (5′-CTTGGTCATTTAGAGGAAGTAA-3′) and VnR (5′-GCTGCGTTCTTCATCGATGC-3′) with barcodes were used to amplify the ITS1 region. The amplification system included 0.3 μM of each primer, 40–60 ng template DNA, 1 μL KOD-FX-Neo (TOYOBO, Osaka, Japan), 1 × buffer and 0.4 mM dNTPs. The amplification conditions were 95°C for 5 min, followed by 15 cycles of 95°C for 1 min, 50°C for 1 min, and 72°C for 1 min, and a final step at 72°C for 7 min (Zang et al., 2018). The amplification products were mixed with VAHTS DNA clean beads (Vazyme, Nanjing, China) for purification, and further amplified by solexa PCR using 0.25 μM of each primer (MPPI-a and MPPI-b), 10 μL purified amplification products, and 1 × Phusion (NEB, Ipswich, Massachusetts). The solexa PCR conditions were 98°C for 30 s, followed by 10 cycles of 98°C for 10 s, 65°C for 30 s, and 72°C for 30 s, and a final step at 72°C for 7 min (Zang et al., 2018). The solexa PCR products were used for DNA library construction after quantification by Qubit 2.0 (Thermo Fisher Scientific, Waltham, MA, United States), mix 1:1 by mass, and DNA gel extraction. Sequencing was performed based on the Illumina HiSeq 2500 platform. The obtained raw sequences were spliced and filtered, and chimeras were removed to generate effective sequences. The lengths of effective sequences were 100–500 bp depending on fungal species, and the average length was 207–284 bp among samples. The raw sequences of six samples analyzed by high throughput sequencing have been submitted to Sequence Read Archive (SRA) database^1^ with BioSample accessions SAMN14766771–SAMN14766776 under the BioProject PRJNA629089. The operational taxonomic units (OTUs) were generated with USEARCH software at a similarity of 97%, and RDP Classifier^2^ with a confidence threshold of 0.8 was used for taxonomic annotation based on the UNITE database^3^. Alpha diversity was analyzed with Chao1, Shannon, and InvSimpson indexes using Mothur software.

### Yeast Isolation and Identification

Each traditional *Xiaoqu* sample was sterilely ground into powder, and 10 g of *Xiaoqu* powder was diluted into 100 mL fluid with distilled water. The fluid with eight sterile glass beads with a diameter of 5 mm added was shaken at 150 r/min and 25°C for 20 min. The decimal dilutions of the fluid were spread on WL nutrient agar (Hopebiol, Qingdao, China) in duplicate and incubated for 5 days at 28°C. WL nutrient agar was used to collect all yeast with different morphologies from *Xiaoqu* samples due to its good identification characteristics (Pallmann et al., 2001). The plates with 30–200 yeast colonies present was selected for morphological observations. The total yeast concentration was quantified as 10^8^–10^9^ cfu/g in each *Xiaoqu* sample. Colonies with different morphologies were selected from the plate, and each single colony was streaked onto new WL nutrient agar for purification and better classification. Pure yeast isolates were cultured in YPD broth (glucose 20 g/L, peptone 20 g/L, and yeast extract powder 10 g/L) at 28°C for 2 days, and further preserved with 20% (volume fraction) glycerol at −80°C.

All yeast isolates were cultured on potato dextrose agar (PDA) (BW-BIO, Shanghai, China) at 28°C for 2 days for further molecular analysis, including 5.8S-ITS-RFLP analysis with *Hae*III restriction enzyme used and D1/D2 sequencing of the 26S rRNA gene. The molecular analysis procedures, including DNA extraction, amplification, digestion, electrophoresis, and sequence alignment, followed those of a previous study by Wang et al. (2019b).

### Micro-Fermentation of Rice Wine With Single Yeast Species

The milled round-grain glutinous rice used for micro-fermentation was purchased from Gaia Farm in northeastern China in 2018. The soluble protein content of the rice was analyzed via the Coomassie brilliant blue method (Cheng, 2014), the starch content was analyzed using the double-wavelength method (Jiang and Hu, 2013), and both were measured with Varioskan Flash (Thermo Fisher Scientific, Waltham, MA, United States). Fat and moisture content could be obtained during the starch analysis, because fat and moisture need to be removed from the rice for an accurate starch analysis.

The rice was developed into hydrolysate medium for micro-fermentation via the following procedures. First, 100 g of rice were soaked in 500 mL distilled water for 10 h then drained. The rice was transferred into a 500 mL triangle flask covered with air permeable sealing film and gauze, then sterilized at 121°C for 20 min. After cooling, 150 mL sterile water, 70 U/g α-amylase (Solarbio Life Sciences, Beijing, China), and 560 U/g glucoamylase (Solarbio Life Sciences, Beijing, China) were added and the solution was stirred then maintained in a water bath at 60°C for 30 min. The pH of the rice hydrolysate medium was 6.5. A 500 mL triangle flask containing rice hydrolysate medium was used for micro-fermentation. The final volume of the rice hydrolysate medium in each triangle flask was 250 mL. Yeast strains maintained as fresh colonies were pre-cultured in 30 mL YPD broth at 28°C overnight with a shaking speed of 120 r/min. A hemocytometer was used under a microscope (Olympus, Tokyo, Japan) to quantify the yeast concentration in the YPD broth. A total of 19 yeast isolates were tested in the micro-fermentations as shown in Tables 2, 3. Each yeast isolate was inoculated into the rice hydrolysate medium at a concentration of 1 × 10^6^ cells/mL. Then, 1 mL of fermentation broth diluted 1000 times after inoculation was spread on WL nutrient agar to check quantification and inoculation accuracy. Fermentations were performed in triplicate for each yeast species at 30°C, and daily weight loss was recorded until not change on weight loss. The fermentation kinetics of 19 yeast isolates were shown in Supplementary Figure S1. The rice hydrolysate medium, after completing fermentation, was filtered with a single layer of gauze. Then, 100 mL filtrate was mixed with 100 mL distilled water and distilled in a 500 mL distilling flask until the distillate reached 100 mL. The distillate was then sealed and stored at 4°C for further higher alcohols and ethanol analysis.

### Gas Chromatograph Analysis of Higher Alcohols and Ethanol

The higher alcohols and ethanol in the distillate of rice wine were analyzed after filtration with a 0.22 μm filter membrane. Analysis was performed with methanol as an external tags using GC-7890A (Agilent Appropriate Technology Co., Ltd., Santa Clara, CA, United States) with a DB-FFAP capillary column (30 m × 0.25 mm × 0.25 μm) and flame ionization detector (Tang et al., 2019). Seven standard substances; ethanol, isoamyl alcohol, β-phenylethanol, isobutanol, propanol, hexanol, and butanol, were used to obtain the retention time and peak area of each substance at eight concentrations, as described by Tang et al. (2019). Further qualitative analysis was conducted according to the retention time, and quantitative analysis was conducted based on the produced standard curves. Temperature procedures were 45°C for 3 min, heating to 120°C at the rate of 16°C/min, maintaining at 120°C for 3 min, heating up to 220°C at the rate of 50°C/min, and holding at 220°C for 5 min. Both the injector port and detector were maintained at 260°C. The flow rates of air, hydrogen, and bypass were separately set as 300 mL/min, 30 mL/min, and 40 mL/min, and the total flow rate detected by GC-7890A was 44 mL/min. A 1 μL sample was retrieved via a micro-syringe with a split ratio of 40:1. A PCA analysis was performed for the 19 yeast isolates using IBM SPSS Statistics 24.0 based on their yields of higher alcohols and ethanol.

## Results

### Fungal Diversity in *Xiaoqu* Samples by High Throughput Sequencing Analysis

**FIGURE 2:**
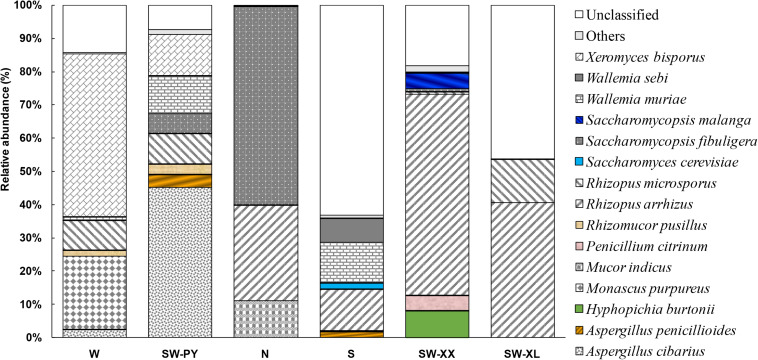
Fungal species with relative abundance higher than 1% in six *Xiaoqu* samples analyzed by high throughput sequencing approach.

High throughput sequencing analysis obtained 55992–75857 effective sequences, and 25–126 observed species among samples (Table 1). The fungal diversity in six *Xiaoqu* samples was evaluated through alpha diversity indexes of high throughput sequencing analysis. SW-XX showed the highest species richness (Chao 1) with 126 species detected, whereas N with Chinese herbs added showed the lowest species richness with 25 species detected. SW-PY exhibited the best species evenness (Shannon and InvSimpson), whereas N showed the worst species evenness. In addition to the differences in alpha diversity among samples, six *Xiaoqu* samples showed variations in the relative abundance of fungal species detected (Figure 2). In total, 15 fungal species were detected with relative abundances greater than 1%; *Aspergillus cibarius*, *Aspergillus penicillioides*, *Hyphopichia burtonii*, *Monascus purpureus*, *Mucor indicus*, *Penicillium citrinum*, *Rhizopus arrhizus*, *Rhizopus microsporus*, *Rhizomucor pusillus*, *S. cerevisiae*, *S’copsis fibuligera*, *Saccharomycopsis malanga*, *Wallemia muriae*, *Wallemia sebi*, and *Xeromyces bisporus* (Figure 2). *R. arrhizus* was the most common fungi specie detected from the six *Xiaoqu* samples, which appeared in four *Xiaoqu* samples with relative abundance higher than 10%. And the next common fungi species were *R. microsporus*, *X. bisporus*, *W. muriae*, and *S’copsis fibuligera* existing in two or three *Xiaoqu* samples with relative abundance higher than 5%. Unclassified species made up a proportion of species present, especially in S (63.1%) and SW-XL (46.0%). Most of the unclassified species could be classified at the genera level, and mainly belonged to the genera of *Aspergillus* and *Wallemia*. The unclassified *Aspergillus* species mainly existed in W (13.8%), S (4.0%), SW-XX (5.2%) and SW-XL (45.9%). And the unclassified *Wallemia* species mainly existed in SW-PY (6.9%), and S (58.8%). Noticeably, over 12% species in SW-XX could not be classified at the genera level, which contained 4.9% species belonging to unknown class of Ascomycota phylum, and 6.0% belonging to unknown phylum.

**TABLE 1:** Alpha diversity indexes of six *Xiaoqu* samples in high throughput sequencing analysis.

*Xiaoqu* samples	Effective sequence numbers	The number of observed species	Chao1	Shannon	Inv- Simpson
W	65503	38	68.33	1.52	3.22
SW-PY	72249	53	58.60	1.86	4.00
N	75857	25	38.75	0.92	2.21
S	55992	63	82.50	1.46	2.62
SW-XX	74421	126	127.25	1.76	2.62
SW-XL	74795	37	46.43	1.02	2.54

Although high throughput sequencing analysis detected rich fungal resources, fewer yeasts were found than filamentous fungi in the six *Xiaoqu* samples. Only four yeast species were found to have a relative abundance higher than 1%; *H. burtonii* and *S. cerevisiae* was found in all samples except W, *S’copsis fibuligera* was found in all samples, and *S’copsis malanga* was only found in S and SW-XX. N with Chinese herbs added showed the highest relative abundance of yeast (59.8% *S’copsis fibuligera*), and W showed the lowest relative abundance of yeast (0.1% *S’copsis fibuligera*). Commercial *Xiaoqu* sample S was made without molding, and showed the highest proportion of *S. cerevisiae* (1.9%) in the six *Xiaoqu* samples.

### Isolation and Identification of Yeasts in Traditional *Xiaoqu* Samples

Although fewer yeasts were directly detected in the five traditional *Xiaoqu* samples by high throughput sequencing analysis, a quantity of yeasts could be isolated on WL nutrient agar. In order to dig out the potential target yeast species, the yeast colonies isolated were further purified and identified by 5.8S-ITS-RFLP analysis and D1/D2 sequencing of the 26S rRNA gene. According to classic culture-dependent approaches, 62 yeast isolates were identified in nine species; *Clavispora lusitaniae* (one isolate), *Cryptococcus neoformans* (one isolate), *H. burtonii* (three isolates), *Komagataella pastoris* (one isolate), *S. cerevisiae* (eight isolates), *S’copsis fibuligera* (32 isolates), *S’copsis malanga* (nine isolates), *Trichosporon asahii* (one isolate), and *Wickerhamomyces anomalus* (six isolates). The sequence details of all isolates are described in Table 2. The nine yeast species showed seven different 5.8S-ITS-RFLP profiles, with the profile between *H. burtonii* and *K. pastoris*, and *S’copsis fibuligera* and *W. anomalus* being the same (Table 2).

**TABLE 2:** Yeast species isolated from *Xiaoqu* samples.

Species/Sources	5.8S-ITS-RFLP analysis		D1/D2 sequencing analysis of 26S rRNA gene		
		
	Size of PCR products (bp)	Size of *Hae*III products (bp)	Isolates designations/GenBank accession number of isolates	GenBank accession number of aligned strains	Identity percentage
*Clavispora lusitaniae*/SW-XL	350	350	FBKL2.8010/MK722465	EF063132	99%
*Cryptococcus neoformans/*SW-PY	600	500 + 120	FBKL2.8025/MK722477	KY107157	99%
*Hyphopichia burtonii*/SW-XX	400	400	FBKL2.8018/MK722470, FBKL2.8021/MK722473, FBKL2.8062/MK722513	U45712	99%
*Komagataella pastoris*/SW-XX	400	400	FBKL2.8064/MK722515	LC090848	100%
*Saccharomycescerevisiae*/SW-XL	840	300 + 220 + 175 + 125	FBKL2.8001-2.8004/ MK722456-722459, FBKL2.8008/MK722463, FBKL2.8009/MK722464, FBKL2.8012/MK722467, FBKL2.8022/MK722474,	AY048154	99%
*Saccharomycopsis fibuligera*/SW-XL, SW-XX, N, SW-PY, W	600	600	FBKL2.8026/MK722478, FBKL2.8027/MK722479, FBKL2.8029-2.8032/MK722481-722484, FBKL2.8034-2.8059/MK722485-722510	U40088	99%
*Saccharomycopsismalanga*/SW-XX	700	700	FBKL2.8015/MK722468, FBKL2.8019/MK722471, FBKL2.8020/MK722472, FBKL2.8028/MK722480, FBKL2.8060/MK722511, FBKL2.8061/MK722512, FBKL2.8063/MK722514, FBKL2.8065/MK722516, FBKL2.8066/MK722517	U40135	99%
*Trichosporon asahii*/SW-XX	500	500	FBKL2.8017/MK722469	AF105393	99%
*Wickerhamomycesanomalus*/SW-XL	600	600	FBKL2.8005-2.8007/ MK722460-722462, FBKL2.8011/MK722466, FBKL2.8023/MK722475, FBKL2.8024/MK722476	EF550341	99%

*Species and isolates marked with underline were further analyzed in micro-fermentations.*

The yeast concentration was 10^8^–10^9^ cfu/g in *Xiaoqu* samples, with N containing the highest yeast concentration (5.30 × 10^9^ cfu/g). *S’copsis fibuligera* was found in all the five traditional *Xiaoqu* samples (Supplementary Table S1); N (12 isolates), SW-PY (11 isolates), SW-XL (4 isolates), W (4 isolates), SW-XX (1 isolates). The other eight yeast species were scattered in different *Xiaoqu* samples; SW-XX contained *H. burtonii*, *K. pastoris*, *S’copsis malanga*, and *T. asahii*, SW-XL contained *C. lusitaniae*, *S. cerevisiae*, and *W. anomalus*, SW-PY contained *C. neoformans*, and *Xiaoqu* samples N and W did not contain any other yeast species except *S’copsis fibuligera*.

### Screening of Yeasts With a Low Yield of Higher Alcohols

The rice used for rice wine fermentations contained 9.21 ± 0.03% (mass fraction) soluble protein, 0.67 ± 0.12% fat, 14.6 ± 0.20% moisture, 0.44 ± 0.15% amylopectin, and 71.70 ± 1.20% amylose. Six yeast species were selected for the micro-fermentation of rice wine, including eight *S. cerevisiae*, six *W. anomalus*, and two *S’copsis malanga* isolates, and one *C. lusitaniae*, *H. burtonii*, and *T. asahii* isolate each (Tables 2, 3). Micro-fermentations inoculated with the 19 isolates showed different fermentation periods (Supplementary Figure S1), ranging from 17 (*C. lusitaniae* FBKL2.8010, *S. cerevisiae* FBKL2.8022, and *W. anomalus* FBKL2.8023) to 40 days (*S. cerevisiae* FBKL2.8003). The total higher alcohol contents in rice wine varied from 50.53 to 277.82 mg/L, depending on the inoculated yeast strain (Table 3). Rice wine fermented by FBKL2.8019 contained the lowest amount of higher alcohols (50.53 mg/L), whereas rice wine fermented by FBKL2.8003 contained the highest amount (277.82 mg/L). Isoamyl alcohol, β-phenylethanol, isobutanol, and propanol are the four primary higher alcohols that were detected in rice wine. Isoamyl alcohol had the highest yield in most rice wines (19.30 mg/L to 120.57 mg/L), followed by β-phenylethanol (19.84 mg/L to 83.95 mg/L), isobutanol (6.84 mg/L to 66.6 mg/L), and propanol (3.75 mg/L to 53.65 mg/L). Most yeast strains produced similar levels of isobutanol and propanol in rice wine, which were nearly half that of isoamyl alcohol. The contents of butanol (not detected to 1.41 mg/L) and hexanol (0.72 mg/L to 9.10 mg/L) were considerably lower (more than 10-fold) than the four primary higher alcohols identified in the rice wine (Table 3).

**TABLE 3:** The amount of higher alcohols and ethanol in rice wine fermented by 19 yeast isolates.

Species/sources	Yeast isolates	Fermentation time (d)	Ethanol (%vol)	Propanol (mg/L)	Isobutanol (mg/L)	Butanol (mg/L)	Isoamyl alcohol (mg/L)	Hexanol (mg/L)	β-phenylethanol (mg/L)	Sum of six higher alcohols (mg/L)
*S’copsis malanga*	FBKL2.8019	22	2.122.45	3.754.51	6.847.94	0.000.00	19.3022.30	0.800.92	19.8422.91	50.5358.42
/SW-XX	FBKL2.8015	19	4.250.19	25.652.65	20.261.38	0.010.01	65.602.53	1.410.05	51.3810.03	164.317.02
*H. burtonii*/SW-XX	FBKL2.8018	26	3.900.12	4.720.72	8.800.92	0.000.00	28.270.86	8.8113.11	29.030.00	79.6313.90
*T. asahii*/SW-XX	FBKL2.8017	22	3.840.18	26.861.39	17.391.09	0.000.00	57.913.54	1.590.35	35.697.72	139.4512.64
*W. anomalus*/SW-XL	FBKL2.8007^#^	34	7.760.37	27.266.23	22.164.46	0.900.52	58.8311.74	2.392.23	46.650.78	158.1820.13
	FBKL2.8024^#^	22	7.322.24	28.987.24	25.842.25	1.100.16	63.1212.63	0.720.46	42.933.59	162.6825.38
	FBKL2.8023^#^	17	7.102.01	30.298.31	27.143.57	1.360.23	61.0211.63	0.900.03	48.947.05	169.6627.30
	FBKL2.8011^#^	38	8.380.13	31.981.94	28.300.59	0.970.05	66.310.97	4.170.11	48.091.68	179.835.03
	FBKL2.8005^#^	38	7.850.18	28.561.58	23.510.31	0.880.07	61.230.95	4.150.76	61.6120.11	179.9520.00
	FBKL2.8006*	39	9.600.59	31.479.76	27.293.24	0.590.30	70.187.43	5.520.33	45.296.93	180.3527.93
*C. lusitaniae/*SW-XL	FBKL2.8010	17	5.220.11	30.394.93	40.081.61	0.410.02	82.723.50	1.120.06	58.162.40	212.8812.09
*S. cerevisiae/*SW-XL	FBKL2.8004^#^	39	8.211.76	38.519.33	40.899.89	0.960.26	79.3617.97	5.280.24	47.371.75	212.3635.81
	FBKL2.8008*	29	10.580.47	43.372.48	46.682.30	1.230.07	95.723.98	6.032.01	46.881.30	239.908.80
	FBKL2.8002^#^	28	9.220.31	30.840.99	46.060.77	0.670.08	81.756.29	0.930.18	83.7222.79	243.9718.51
	FBKL2.8009*	39	10.230.14	49.002.26	46.800.66	1.320.03	92.191.93	5.740.50	49.320.89	244.372.95
	FBKL2.8001*	36	9.911.14	33.672.58	53.0110.56	0.790.16	95.3716.05	1.140.08	72.9817.50	256.9523.92
	FBKL2.8012*	38	11.290.35	53.654.38	50.922.66	1.410.20	102.725.75	6.500.70	42.334.25	257.538.07
	FBKL2.8022*	17	11.700.19	34.991.80	66.602.81	0.890.11	120.572.29	0.940.05	53.771.06	277.754.81
	FBKL2.8003*	40	9.960.23	46.481.03	46.615.51	1.120.03	90.576.28	9.100.36	83.958.36	277.8220.56

* Strains with ethanol yields greater than 10%, ^#^Strains with ethanol yields greater than 7% but lower than 10%.

The ethanol contents in rice wine ranged from 2.12% to 11.70% (volume fraction), as shown in Table 3. Six *S. cerevisiae* strains (FBKL2.8001, FBKL2.8003, FBKL2.8008, FBKL2.8009, FBKL2.8012, and FBKL2.8022) and one *W. anomalus* strain (FBKL2.8006) were high ethanol producers with yields greater than 10%. Two *S. cerevisiae* (FBKL2.8002 and FBKL2.8004) and five *W. anomalus* strains (FBKL2.8005, FBKL2.8007, FBKL2.8011, FBKL2.8023, FBKL2.8024) were medium ethanol producers with yields greater than 7%, but lower than 10%.

**FIGURE 3:**
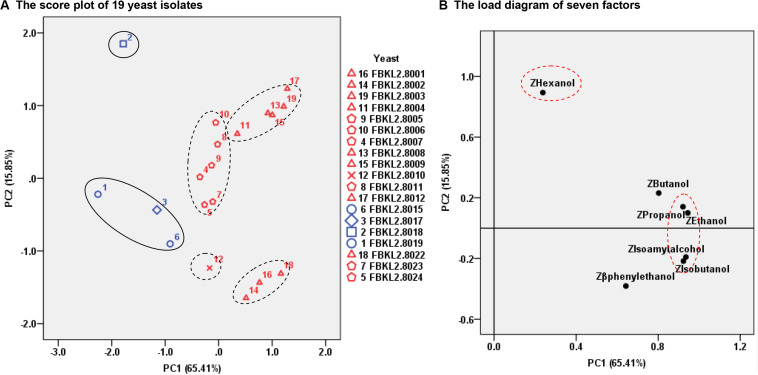
PCA analysis of the 19 yeast isolates based on their yields of higher alcohols and ethanol in rice wine fermentation. **(A)** The score plot of 19 yeast isolates. The four yeast isolates with tags of 1, 2, 3, and 6 originated from the *Xiaoqu* sample SW-XX. The other 15 yeast isolates originated from the *Xiaoqu* sample SW-XL. **(B)** The load diagram of seven factors.

The PCA analysis differed the 19 yeast isolates into five groups, and the yeast isolates with different origins and identities were well differentiated under the influence of PC1 and PC2 (Figure 3A). PC1 was mainly affected by the contents of ethanol, isoamyl alcohol, propanol and isobutanol, and PC2 was mainly affected by the hexanol content (Figure 3B). Eight *S. cerevisiae* were separated into two groups under the influence of PC2, with FBKL2.8001, FBKL2.8002, and FBKL2.8022 being one group containing less hexanol content than the other groups with five isolates.

## Discussion

Increasing attention is being given to maintaining a high standardized quality of baijiu and other traditional Chinese alcoholic beverages such as rice wine, which requires a standardized and well-controlled production process. Therefore, in recent years, microbial diversity analyses in *Jiuqu*, the production environment, and fermentation processes have been undertaken to investigate microbial resources and potential beneficial microorganisms (Jin et al., 2017). The screened microorganisms are proposed to be safe and effective starters, as opposed to traditional starters, to promote the standardization of production (Cai et al., 2018). As well as a main baijiu and rice wine production region in China, Guizhou also traditionally produces *Xiaoqu*. To the best of our knowledge, this study, for the first time, analyzed fungal diversity among traditional *Xiaoqu* samples from different regions of Guizhou in China and further screened fermentative yeast with application value in the production of baijiu and rice wine.

Filamentous fungi were the primary fungi identified in six different *Xiaoqu* samples in this study, and several yeast species were detected with a relative abundance greater than 1%, with unclassified fungi taking up a proportion of these species. These findings were consistent with those of previous studies regarding fungal diversity in *Xiaoqu* samples from different regions of Southern China, including provinces of Hubei, Jiangsu, Zhejiang, Guangxi, Sichuan, and East Guizhou (Gou et al., 2015; Wu et al., 2017; Cai et al., 2018). However, fungal diversity in *Xiaoqu* varied depending on the different geological locations. This study showed the obvious fungal diversity variation among *Xiaoqu* from six different regions of Guizhou province, even if two of the samples were from the same Country in the southwestern region of Guizhou. *R. arrhizus* was the most common filamentous fungi specie in *Xiaoqu* samples found in this study with the relative abundance of 28.8% in N, 12.5% in S, 40.7% in SW-XL, and 60.4% in SW-XX, whereas a dominant occurrence of this species or genera was reported in *Xiaoqu* from Sichuan province (80.6%, Gou et al., 2015), and *Xiaoqu* from Jiangsu, Zhejiang, Guangxi, and Guizhou provinces (higher than 95%, Cai et al., 2018). In addition, *Xiaoqu* from neighboring provinces of Guizhou, also showed the variation of relative abundance of main fungal species, such as 80.6% of *R. oryzae* and 19.3% of *S’copsis fibuligera* in *Xiaoqu* from Luojiang Country of Sichuan province, 94.9% of *R. stolonifer* in *Xiaoqu* from Pengzhou Country of Sichuan province, and 67.4% of *C. bertholletiae*, 18.9% of *R. stolonifer*, and 10.9% *S. cerevisiae* from Zigui Country of Hubei province (Gou et al., 2015; Wu et al., 2017; Cai et al., 2018). In addition to the difference of main fungal species, higher fungal species richness was exhibited in traditional Guizhou compared to other reported *Xiaoqu*. Both high throughput sequencing and classic culture-dependent approaches detected new fungal species that had not been reported in previous studies (Wu, 2004; Li and Fang, 2007; Gou et al., 2015; Jin et al., 2017; Wu et al., 2017; Cai et al., 2018), containing eight filamentous fungi species (*M. purpureus*, *P. citrinum*, *R. arrhizus*, *R. microsporus*, *R. pusillus*, *W. muriae*, *W. sebi*, and *X. bisporus*) and six yeast species (*C. lusitaniae*, *C. neoformans*, *H. burtonii*, *K. pastoris*, *S’copsis malanga*, and *T. asahii*).

The dissimilar fungal diversity among *Xiaoqu* in different geological locations might correlate with the production microhabitats of *Xiaoqu*, especially for the Qu house (Wang et al., 2020). Furthermore, the production technology of *Xiaoqu* such as the use of different raw materials and molding methods might also play a role in forming different fungal diversity (Wang et al., 2018). N had a variety of Chinese herbs added and exhibited the lowest species richness and evenness as well as the lowest proportion of unclassified species. N also showed the highest relative abundance of yeast (59.8% *S’copsis fibuligera*). These findings confirmed the potential effect of Chinese herbs on microorganisms, of decreasing fungal diversity and promoting yeast growth (Wang et al., 2019a). Commercial *Xiaoqu* sample S had no molding (*Sanqu*) and showed the highest proportion of unclassified species (63.1%, mainly from the genera of *Aspergillus* and *Wallemia*) and *S. cerevisiae* (1.9%). These results differed from those of Wang et al. (2018) who found that traditional *Xiaoqu* showed higher bacterial diversity than *Sanqu*.

The combination of high throughput sequencing and culture-dependent approaches helped to dig out the rich fungal resources in *Xiaoqu* in this study. The high throughput sequencing especially contributed to a comprehensive recognition of fungal diversity. The occurrence ratios of *H. burtonii*, *S’copsis fibuligera*, and *S’copsis malanga* analyzed by culture-dependent approaches coincided with their relative abundance in high throughput sequencing analysis. *S. cerevisiae* was found with relative abundance lower than 1% in N, SW-PY, SW-XL, and SW-XX by high throughput sequencing analysis, whereas it was only isolated from SW-XL. The isolation and identification of new fungal species are beneficial for enriching fungal sequence databases, and thus, reducing the proportion of unclassified fungal species in the analysis of high throughput sequencing (Wang et al., 2019b). The unclassified species from the genera of *Aspergillus* and *Wallemia*, and from the unknown phylum or the unknown class of Ascomycota are needed to further analyze to explore the specific fungal resources in traditional Chinese *Xiaoqu*.

The content of higher-alcohols in California red wine was 140–417 mg/L (Zhu et al., 2016), in baijiu was about 600–1200 mg/L (Jin et al., 2017), and in European spirits was 0–10000 mg/L with the mean value of 4000 mg/L (Lachenmeier et al., 2008). Attempts have been made to reduce the amount of higher alcohols because of the potential health hazards and bitter taste caused by excess amount (Yan et al., 2012). This study for the first time evaluated the higher alcohols yields of wild yeast species in traditional *Xiaoqu* from Guizhou province, including two common yeast species in *Xiaoqu*, *S. cerevisiae* and *W. anomalus*, as well as four new yeast species, *C. lusitaniae*, *H. burtonii*, *S’copsis malanga*, and *T. asahii*. All yeast strains produced lower levels of higher alcohols in rice wine than other reported isolates (0.34 g/L to 3.98 g/L) (Wang et al., 2019a). However, the raw materials used are different in these studies including glutinous rice, corn, sorghum and malt extract medium, which contain different levels of amino acids, and thus, affect the yield of higher alcohols. Therefore, comparisons between previous studies and the present study would be improved if the same raw materials are used for fermentations of these isolates in future studies (Wang et al., 2019a). Moreover, *S. cerevisiae* ARTP5 obtained from mutagenesis has been found to produce 7.27 mg/L propanol, 14.14 mg/L isobutanol, and 44.99 mg/L isoamyl alcohol in fermented glutinous rice (Wang et al., 2019a). The laboratory *S. cerevisiae* ARTP5 produced less higher alcohols than the *S. cerevisiae* isolates from traditional *Xiaoqu* in Guizhou. Although the four new yeast species, *C. lusitaniae*, *H. burtonii*, *S’copsis malanga*, and *T. asahii* could produce less higher alcohols than most *S. cerevisiae* reported, their ethanol-producing ability was quite limited (lower than 5.5%) which could not meet the demands of rice wine and baijiu. *S. cerevisiae* and *W. anomalus* displayed relatively strong ethanol-producing abilities of greater than 7%, especially for *S. cerevisiae* strains, as reported by Fleet (2008). Therefore, *S. cerevisiae* FBKL2.8022 was screened as a fermentative yeast due to its high ethanol-producing ability (11.70%), fast fermentation speed (17 days), and low yield of higher alcohols (277.75 mg/L). *W. anomalus* FBKL2.8023 has also been screened for making rice wine with medium levels of ethanol content as a result of its medium ethanol-producing ability (7.10%), fast fermentation speed (17 days), and low yield of higher alcohols (169.66 mg/L). In addition, isoamyl alcohol and isobutanol were reported to be the two main higher alcohols in baijiu inoculated with *Xiaoqu*, the former taking up 45–65% of the content of total higher alcohols (Yan et al., 2012). However, the rice wines fermented by 19 yeast isolates in this study showed a high content of β-phenylethanol, which was similar to isoamyl alcohol in some cases, and the content of propanol was also similar to isobutanol in this study.

*W. anomalus* is capable of producing phenylethyl acetate by the reaction of phenylethanol and acetic acid during fermentation (Liu et al., 2015). Our initial analysis demonstrated that *W. anomalus* ranked first on the tests of ester-producing ability in all the 66 yeast isolates (data not shown). Further studies should analyze the production ability of phenylethyl acetate of *W. anomalus* strains isolated from traditional Guizhou *Xiaoqu* samples, considering its phenylethanol yield. *S’copsis fibuligera* was the common yeast species in *Xiaoqu* and was largely isolated in this study. However, no isolates of *S’copsis fibuligera* were inoculated for micro-fermentations in this study, mainly because it was well known for the amylolytic ability instead of ethanol-producing ability (Cai et al., 2018). In addition, the colony and cell morphology of *S’copsis fibuligera* was similar to filamentous fungi, as well as the new yeast species of *C. neoformans* and *K. pastoris*, the ethanol-producing ability and higher alcohols yields of which were also not tested in this study. The ester-producing ability of the three yeast species were initially found lower than *W. anomalus* that we screened in this study (data not shown). Future studies will consider to use the mix inoculation of amylolytic, fermentative, and ester-producing fungal species to improve the aroma complexity of rice wine and baijiu.

In summary, traditional Chinese *Xiaoqu* samples showed different fungal diversity compared to another and the results of previous studies. The present study displayed distinct fungal resources for screening advantageous fungal species and producing standardized *Xiaoqu*. *S. cerevisiae* FBKL2.8022 and *W. anomalus* FBKL2.8023 were selected as fermentative yeasts with low yields of higher alcohols and high or medium ethanol-producing abilities. The amylolytic ability of fungal species, such as *R. arrhizus*, *R. microsporus*, *S’copsis fibuligera*, and *S’copsis malanga*, will be evaluated in future studies for mixed inoculation with the two yeast species screened. These studies will investigate the potential for their application in the production of baijiu and rice wine of high quality.

## Data Availability Statement

The datasets presented in this study can be found in online repositories. The names of the repository/repositories and accession number(s) can be found in the article/ Supplementary Material.

## Author Contributions

CW designed and performed the experiments and wrote the article. JT performed the experiments. SQ designed the article. All authors contributed to the article and approved the submitted version.

## Conflict of Interest

The authors declare that the research was conducted in the absence of any commercial or financial relationships that could be construed as a potential conflict of interest.

## Footnotes

1 https://submit.ncbi.nlm.nih.gov/subs/sra/

2 http://sourceforge.net/projects/rdpclassifier/

3 http://unite.ut.ee/index.php

## Supplementary Material

FIGURE S1 The kinetic of micro-fermentation inoculated with 19 yeast isolates.

TABLE S1 The details of *Xiaoqu* samples.
