# Supplementary figures and images for: Profiling of Fungal Diversity and Fermentative Yeasts in Traditional Chinese Xiaoqu
Source: Front Microbiol. 2020 Aug 31;11:2103. doi: 10.3389/fmicb.2020.02103 (PMC7489096; doi:10.3389/fmicb.2020.02103)

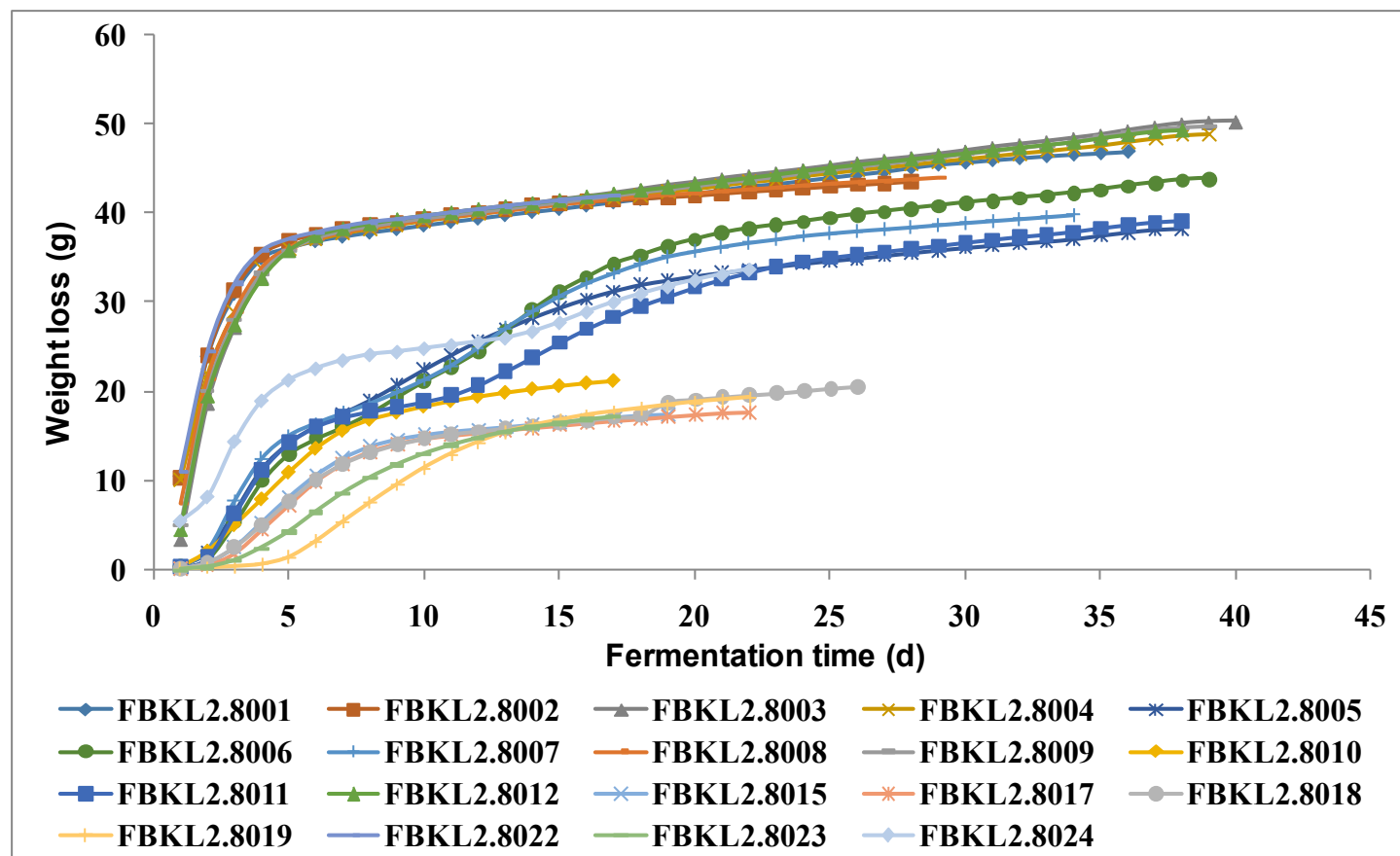

Supplementary Figure 1 The kinetic of micro-fermentation inoculated with 19 yeast isolates.

Supplement: FIGURE S1 — The kinetic of micro-fermentation inoculated with 19 yeast isolates. [file Image_1.pdf]
