# Supplementary material for: Profiling of Fungal Diversity and Fermentative Yeasts in Traditional Chinese Xiaoqu
Source: Front Microbiol. 2020 Aug 31;11:2103. doi: 10.3389/fmicb.2020.02103 (PMC7489096; doi:10.3389/fmicb.2020.02103)
Supplement: TABLE S1 — The details of Xiaoqu samples. [file Table_1.docx]

Supplementary Table 1 The details of *Xiaoqu* samples

| *Xiaoqu* samples | The location of *Xiaoqu* | The shape of *Xiaoqu* | The special part of *Xiaoqu*-making process | The number of yeast isolates | Yeast species |
| --- | --- | --- | --- | --- | --- |
| N | Zunyi City located in the northern region of Guizhou | Quadrate with a length of 0.04 m | With Chinese herbs added | 12 | *Saccharomycopsis fibuligera* |
| SW-PY | Panzhou Country located in the southwestern region of Guizhou | Cake-like with a diameter of 0.06 m and thickness of 0.02 m | No specialty | 1 | *Cryptococcus neoformans* |
| SW-XL | Liguan of Xingren Country located in the southwestern region of Guizhou | Cake-like with a diameter of 0.07 m and a thickness of 0.02 m | No specialty | 1 | *Clavispora lusitaniae* |
|  |  |  |  | 8 | *Saccharomyces cerevisiae* |
|  |  |  |  | 4 | *Saccharomycopsis fibuligera* |
|  |  |  |  | 6 | *Wickerhamomyces anomalus* |
|  |  |  |  | 11 | *Saccharomycopsis fibuligera* |
| SW-XX | Xiaojiawan of Xingren Country located in the southwestern region of Guizhou | Lump-shaped with a length of 0.02–0.04 m | No specialty | 3 | *Hyphopichia burtonii* |
|  |  |  |  | 1 | *Komagataella pastoris* |
|  |  |  |  | 1 | *Saccharomycopsis fibuligera* |
|  |  |  |  | 9 | *Saccharomycopsis malanga* |
|  |  |  |  | 1 | *Trichosporon asahii* |
| W | Weining Country located in the western region of Guizhou | A sphere with a diameter of 0.07 m | No specialty | 4 | *Saccharomycopsis fibuligera* |
| S | Qiannan located in the southern region of Guizhou | A pile of debris | Commercial *Xiaoqu* without molding | Yeast isolation was not performed. | |
